# 

**Published:** 1853-08

**Authors:** 


### CONTENTS.

ORIGINAL COMMUNICATIONS:— PAGE

ART. I.—Milksickness. By Samuel Thompson, M.D. . . . 225
ART. II,—Castration, etc. By A. H. Van Norstrand, M.D. . . 234
ART. III.—A Few Thoughts, etc. By Chas. L. Anderson, M.D. · 236
ART IV.—The Success of Homœopatic Practice. By De Laskie Miller 239
ART. V.—An Inquiry, Critical and Experimental, etc. By N. S. Davis, M.D. . . . . . . . . . . . . . . 241

SELECTIONS:—

Neuralgic Operations, . . . . . . . . . . . . 257
Anœsthetic Properties of the Lycoperdon Proteus, . . . . . . 258
Treatment of Chronic Abscesses of the Breast, etc. . . . . . 260
Hot Water and Soap in Ptyalism, . . . . . . . . . 263
Case of Traumatic Tetanus cured by the Inhalation of Chloroform, . 264
The Effects of Different Remedies in Diabetes, . . . . . . 265
Lactine as an Article of Nutrition, . . . . . . . . 267
On the Utility of administering Opium by the Urethra, . . . . . 268
Fœtus in Utero, killed by Lightning, . . . . . . . . 269
Chlorate of Potash in Ulcerative Stomatitis, etc. . . . . . . 269
Chloride of Soda, . . . . . . . . . . . . 270
Collodion for Chordee, . . . . . . . . . . 270
Fly Poison . . . . . . . . . . . . . . . 271
Deaths by Chloroform. . . . . . . . . . . . 271
Treatment of Ulcers, Death of Dr. Beaumont, External Use of Ipecac, 272

EDITORIAL:—

Medical Society of the State of Wisconsin, . . . . . . . 273
The Wisconsin Medical Association, . . . . . . . . 274
La Salle County Medical Society, . . . . . . . . . . 275
Cook County Medical Society, . . . . . . . . . . 276
The Influence of the Practice of the Daguerrotype Art, . . . . 279
The American Association for the Advancement of Science, . . . . 280
Death of Pereira, . . . . . . . . . . . . 287
Atlas of Pathological Histology, . . . . . . . . . 288

